# The Role of NQO1 in Ovarian Cancer

**DOI:** 10.3390/ijms24097839

**Published:** 2023-04-25

**Authors:** Giovanni Tossetta, Sonia Fantone, Gaia Goteri, Stefano Raffaele Giannubilo, Andrea Ciavattini, Daniela Marzioni

**Affiliations:** 1 Department of Experimental and Clinical Medicine, Università Politecnica delle Marche, 60126 Ancona, Italy; g.tossetta@univpm.it (G.T.); s.fantone@pm.univpm.it (S.F.); 2 Department of Biomedical Sciences and Public Health, Università Politecnica delle Marche, 60126 Ancona, Italy; g.goteri@staff.univpm.it; 3 Department of Clinical Sciences, Università Politecnica delle Marche, Salesi Hospital, 60123 Ancona, Italy; s.r.giannubilo@staff.univpm.it (S.R.G.); a.ciavattini@univpm.it (A.C.)

**Keywords:** NQO1, ovarian cancer, NAD(P)H:quinone oxidoreductase 1, compounds, natural, synthetic, antioxidants, NRF2, pathway, signalling

## Abstract

Ovarian cancer is one of the most dangerous gynecologic malignancies showing a high fatality rate because of late diagnosis and relapse occurrence due to chemoresistance onset. Several researchers reported that oxidative stress plays a key role in ovarian cancer occurrence, growth and development. The NAD(P)H:quinone oxidoreductase 1 (NQO1) is an antioxidant enzyme that, using NADH or NADPH as substrates to reduce quinones to hydroquinones, avoids the formation of the highly reactive semiquinones, then protecting cells against oxidative stress. In this review, we report evidence from the literature describing the effect of NQO1 on ovarian cancer onset and progression.

## 1. Introduction

**Figure 1:**
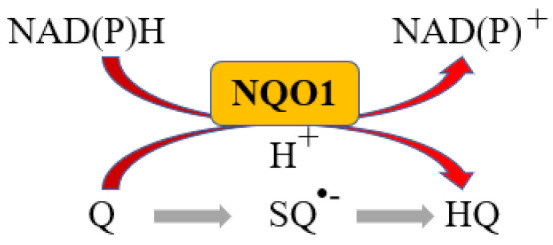
NQO1 reaction. Q: Quinone, SQ^•−^: Semiquinone, HQ: hydroquinone.

More than 95% of all ovarian cancers (OC) are epithelial (EOC) [1] and can be classified, according to histological characteristics, into five subtypes: high-grade serous carcinoma (HGSOC; 70%), endometrioid carcinoma (10%), clear-cell carcinoma (5–10%), low-grade serous carcinoma (less than 5%), and mucinous carcinoma (around 3%) [2]. The NAD(P)H:quinone oxidoreductase 1 (NQO1) is an antioxidant enzyme which uses NADH or NADPH as substrates to reduce quinones to hydroquinones avoiding the formation of the highly reactive semiquinones, then protecting cells against oxidative stress [3] (Figure 1). 

Although NQO1 can protect normal cells from oxidative stress, it has been reported that a high expression of NQO1 at the early stages of carcinogenesis can favor cancer cell growth [4,5,6]. The clinical research is always focused on finding novel biomarkers for prognostic purposes as well as therapeutic targets since they are of crucial importance to improve the outcome of patients affected by aggressive neoplasms [7,8,9,10,11,12,13]. It has been reported that NQO1 is highly expressed in many solid tumors such as uterine cervix [14], endometrium [14], lung [15] colon [16], pancreas [17] and ovarian cancer [18]. NQO1 expression is significantly higher in ovarian carcinoma compared to normal and precancerous lesions, suggesting that NQO1 may be a therapeutic target in ovarian cancer [19]. 

Cui and colleagues reported that NQO1 protein was predominantly expressed in the cytoplasm of ovarian carcinoma cells. Moreover, the authors confirmed that NQO1 expression was significantly higher in ovarian carcinoma compared to borderline serous tumors or benign serous tumors. NQO1 expression in borderline serous tumors was also higher than in benign serous tumors. Interestingly, the authors determined that the high expression of NQO1 protein was associated with higher histological grade, advanced clinical stage and lower overall survival in ovarian carcinomas. Moreover, multivariate analysis showed that, in addition to clinical stage, NQO1 expression was a significant independent prognostic factor. Thus, NQO1 expression could be an important biomarker for poor prognostic evaluation in patients with serous ovarian carcinomas [20]. 

Estrogens can significantly promote ovarian cancer development stimulating ovarian surface epithelial cells proliferation and invasiveness [21] since both nuclear estrogen receptors (ER) α and β are expressed in the majority of ovarian cancer [22,23,24]. Estrogens can be formed from the inactive steroid precursor estrone sulfate (E1-S) by the steroid sulfatase (STS) enzyme or from dehydroepiandrosterone sulfate (DHEA-S) or androstenedione by the aromatase (CYP19A1) enzyme [25]. An interesting study by Pavlic and colleagues reported a high steroid sulfatase expression and weak CYP19A1 expression in OVSAHO, Kuramochi, COV632 (three HGSOC cell lines) compared to immortalized normal ovarian epithelial HIO-80 cells, indicating that these cells produce estrogens from the precursor estrone sulfate (E1-S). Among ovarian cancer cells, the metabolism of E1-S to estradiol was the highest in OVSAHO, followed by Kuramochi and COV362 cells. Moreover, NQO1 and Glutathione S-transferase P1 (GSTP1) expression was significantly higher in COV362 cells compared to OVSAHO cells. Thus, the lower formation of estrogens observed in the COV362 cells compared to the OVSAHO cells may be associated with the differences in the oxidative metabolism of estrogens in these cells. In fact, COV362 cells form higher levels of oxidate Estrone (E1)/Estradiol (E2) (due to the increased NQO1 expression) and glutathione-conjugated quinones (due to the increased GSTP1 expression). Then, this study suggests that the difference in estrogen metabolism in the four HGSOC subtypes may, at least in part, be due to differential expression of NQO1 in these cells [26].

NADPH and oxidative stress are regulated by xanthine oxidoreductase (XOR), the activity of which can be measured by uric acid levels, a metabolite associated to the development of cardiovascular disease [27,28], which is a great burden for oncologic patients. Thus, it is possible that NQO1 and XOR influence each other, suggesting that NQO1 may act on not only the prognosis of ovarian cancer, but also the cardiovascular burden associated with ovarian cancer [29].

The aim of this review is to provide an overview of the current literature regarding the role of NQO1 in ovarian cancer onset and progression with a focus on its cellular modulators and targets.

## 2. Role of NQO1 Polymorphisms in Ovarian Cancer

The term polymorphism refers to the presence in two or more variant forms of a specific DNA sequence in different individuals. Single-nucleotide polymorphisms (SNPs) are the most common type of polymorphisms that involve variations in a single nucleotide [30]. It is known that the presence of SNPs within the coding regions of genes can modify the amino acid sequence of the encoded products causing non-synonymous substitutions that can alter protein structure, then impairing its function [31,32]. However, it has been reported that the presence of SNPs in the 5′ or 3′ untranslated regions (UTR) of genes can also have significant effects on gene expression since SNPs can affect regulatory elements or mRNA stability, impairing gene expression. In fact, the presence of SNPs at 5′-UTR can interfere with mRNA splicing, regulation of transcription (e.g., altering methylation) or translation (e.g., altering the ribosome binding site) [32,33,34]. Gene expression can also be altered by the presence of SNPs at 3’-UTR of mRNA since SNPs can alter the poly-(A) tail, which protects mRNA molecule from exonucleases degradation. In addition, SNPs can alter mRNA expression, modifying the microRNA-binding sites, which can be present at 5′-, 3′-UTRs and in the coding region of mRNA [32,35,36]. 

NQO1 gene expression is induced under stimuli of xenobiotics, antioxidants, radiotherapy and chemotherapeutic agents (e.g., platinum-based drugs). Moreover, under oxidative stress, NQO1 can bind and stabilize mutant and wild-type p53, an important tumor suppressor, inhibiting its degradation [37].

Three principal SNPs in NQO1 gene have been studied in ovarian cancer: rs1800566, rs1131341 and rs2917666. The characteristics and locations of these SNPs are reported in Table 1. 

NQO1 SNP rs1800566 is a c.558C >T missense variant in exon 6 of the NQO1 gene which causes the substitution of proline for serine (P > S), leading to a reduction in the activity of NQO1 enzyme in C/T heterozygotes while causing a NQO1 inactivity in T/T homozygotes [38,39,40] resulting in an increased risk of ovarian cancer. Although all these variants have been detected in both cancer and control samples, the authors did not report a significant prevalence of these SNPs in patients with ovarian cancer, suggesting that these variations of NQO1 may not be linked to ovarian cancer development [41].

BReast CAncer gene 1 and 2 (BRCA-1, -2) are two tumor suppressor genes involved in DNA repair. Therefore, the inheritance of a single BRCA1 or BRCA2 mutation leads to a high risk to develop ovarian cancer during lifetime [42]. An interesting study evaluated the relevance of SNP rs1800566 in NQO1 gene in a population carrying BRCA1/BRCA2 mutation. The authors detected no differences in the incidence of rs1800566 polymorphism between the patients with ovarian cancer and healthy subjects [43]. 

To date, platinum drugs such as cisplatin, carboplatin, and oxaliplatin are the most used clinical agents in chemotherapy against ovarian cancer. However, although these drugs show a good efficiency at beginning of therapy, many of the patients relapse within 18 months due to chemoresistance [18,44]. Thus, the clinical availability of specific makers that can efficiently and accurately predict chemotherapy responses might significantly improve cancer outcomes in these patients.

NQO1 SNP rs1131341 is a C > T variant of the NQO1 gene in the exon 4 of the NQO1 gene, which causes the substitution of arginine at position 139 for tryptophan. An interesting study evaluated the role of SNPs rs1131341 and rs1800566 in NQO1 gene in patients with ovarian cancer receiving cisplatin/cyclophosphamide chemotherapy. The authors of the study reported that both SNPs in NQO1 gene were significantly associated with progression-free survival (PFS). However, this difference became not significant after adjustment for variations in treatment. Although SNPs presence in NQO1 gene could be involved in drug metabolism, studies involving more patients are needed to identify patients at risk for nonresponse to cisplatin-based treatment [45]. 

NQO1 SNP rs2917666 is a C > G variant of the NQO1 gene in the 3′ untranslated region (UTR) of the NQO1 gene, and the presence of this SNP in NQO1 gene has been significantly associated with invasive epithelial ovarian cancer. Thus, the presence of this SNP may impair NQO1 function favoring carcinogenesis [46].

Depending on the position of the SNP in the NQO1 gene, the expression/activity of NQO1 can be altered due to the substitution of a specific amino acid in the catalytic site of the enzyme (e.g., the SNPs rs1800566 and rs1131341) or to the altered expression of its mRNA (e.g., the SNP rs2917666). The impaired expression/activity of NQO1 enzyme in normal cells makes them vulnerable to oxidizing agents favoring carcinogenesis. Moreover, SNPs in the UTR regions can favor the expression of NQO1 mRNA, making cancer cells resistant to chemotherapeutics agents (especially to the platinum-derived ones). 

Looking at the studies discussed in this section, we can conclude that both rs1131341 and rs1800566 SNPs in NQO1 gene were not associated to ovarian cancer onset and/or progression-free survival (PFS), while rs2917666 was associated to invasive epithelial ovarian cancer.

## 3. NQO1 Cellular Modulators in Ovarian Cancer

Hypoxia is involved in several diseases, and hypoxia-inducible factor-1 (HIF-1) is a key factor modulated by this process. HIF-1 consists of two subunits: alpha subunit (HIF-1α) and beta subunit (HIF-1β). HIF-1α expression is regulated by oxygen tension while HIF-1β is constitutively expressed. HIF-1α plays a key role in modulating many cellular processes such as angiogenesis, cell proliferation, invasion and tumor progression [47,48,49,50]. Moreover, it has been reported that overexpression of HIF-1α in solid tumor can compromise chemotherapy. In fact, the increased expression of HIF-1α is due to the stabilization effect of NQO1 that inhibits proteasome-mediated degradation of HIF-1α interacting with the oxygen-dependent degradation (ODD) domain of HIF-1α [51]. 

Interestingly, Wang and colleagues determined that hypoxia-responsive polymer micelles such as methoxyl poly (ethylene glycol)-co-poly(aspartate-nitroimidazole) synergically act with a NQO1 inhibitor (dicoumarol) to sensitize SKOV3 ovarian cancer cell line to the anticancer agent sorafenib under low oxygen conditions. In fact, treatment of SKOV3 with hypoxia-responsive micelles containing sorafenib and dicoumarol significantly reduced NQO1 activity compared to drug-free micelles. This reduced NQO1 activity was due to the presence of dicoumarol, which competes with NAD(P)H (a cofactor of NQO1) for binding to the oxidized form of NQO1, and to the micelles polymer that reduces NAD(P)H levels. Interestingly, the authors determined that the expression of HIF-1α was significantly repressed in cells treated with micelles loaded with dicoumarol, and the degradation of HIF-1α significantly increased the vulnerability of SKOV3 cells to sorafenib in cells treated with micelles loaded with sorafenib and dicoumarol, leading to an increased apoptosis. Thus, this study demonstrates that NQO1 plays a key role in HIF-1α stabilization, and a dual treatment with dicoumarol and sorafenib can significatively increase sorafenib sensitivity in ovarian cancer cells [52]. 

Looking at these studies [51,52], we can conclude that the high levels of NQO1 in ovarian cancer cells play a key role in stabilizing HIF-1α under hypoxia, an important factor characterizing tumor microenvironment [53], favoring HIF-1α-mediated processes such as tumor angiogenesis and cancer cell proliferation. Thus, inhibition of NQO1 may be an efficient therapeutic strategy to inhibit ovarian cancer progression. 

Rgnef (ARHGEF28/p190RhoGEF) is a Rho-specific guanine nucleotide exchange factor (GEF) that is activated downstream of integrins and favors RhoA GTPase activation and actin stress fiber formation, then promoting cell migration [54,55]. It has been reported that Rgnef protein expression was significantly increased in patients with late-stage serous ovarian cancer and high Rgnef levels were associated with decreased progression-free and overall survival of these patients. Moreover, knockout of Rgnef in aggressive murine ID8-IP cell line significantly decreased NQO1 expression, suggesting that Rgnef can regulate oxidative stress levels modulating NQO1 expression [56].

Nuclear Factor Erythroid 2-Related Factor 2 (NFE2L2 or NRF2)/Kelch Like ECH Associated Protein 1 (KEAP1) signalling is one of the most important pathways involved in chemoresistance onset and cancer progression [14,57,58]. In fact, NRF2 can bind the antioxidant response element (AREs) regions present in the promoter of several antioxidant genes such as NQO1, superoxide dismutase (SOD), catalases (CATs), thioredoxins (Trxs), peroxiredoxins (Prxs), reductases and peroxidases inducing their expression and counteracting the oxidant effects of platinum-based chemotherapeutics, then leading to chemoresistance in these cells [14,57,59,60,61].

Long non-coding RNAs (lncRNAs) are RNA sequences which do not code any polypeptide or protein and act by sponging and sequestering micro RNAs (miRNAs). Both miRNAs and lncRNAs play a key role in cancerous and non-cancerous diseases [62,63,64,65,66]. LncRNA H19 is encoded by the H19 gene and plays a key role in cancer onset and progression modulating cell growth, invasion and migration [67]. An interesting study reported that lncRNA H19 levels were significantly increased in cisplatin-resistant A2780/CDDP ovarian cancer cells and in patients with high-grade serous ovarian cancer (HGSC). Furthermore, NQO1 expression was significantly higher in A2780/CDDP compared to that of the parental cell line A2780 (cisplatin sensitive). Interestingly, knockdown of lncRNA H19 in A2780/CDDP cells restored cisplatin sensitivity reducing NQO1 and NRF2 expression, proving that lncRNA H19 has a pivotal role in cisplatin resistance of ovarian cancer cells modulating NRF2/NQO1 signaling [68].

This expression pattern of NQO1 and NRF2 was also reported by Bao and colleagues, who detected an increased expression of NQO1 and its regulator NRF2 in cisplatin-resistant ovarian cancer cells A2780/CDDP compared to cisplatin-sensitive parental cell line A2780. Moreover, they discovered that silencing of NRF2 sensitized A2780/CDDP cells to cisplatin treatment, decreasing NQO1 protein expression and increasing cisplatin-induced cell death, demonstrating a key role of NRF2/NQO1 signaling in the development of cisplatin resistance [69]. 

The studies discussed in this section are summarized in Table 2.

## 4. NQO1 Modulation by Natural and Synthetic Compounds in Ovarian Cancer

Natural compounds (also known as phytonutrients) are biological compounds that can be found in plants, bacteria, fungi and marine organisms. These compounds are often used as diet supplement worldwide showing important antioxidants, anti-inflammatory and anti-cancer effects [70,71,72,73,74,75]. 

Vitamin K3, also known as menadione, is a synthetic derivative of vitamin K with high biological activity that can also be produced in the body through metabolic conversion of vitamin K1 (phylloquinone) [76,77].

It has been reported that vitamin K3 may have potential anti-tumor effects promoting ROS production [78]. In fact, Xia and colleagues determined that cisplatin-resistant ovarian cancer cell line SKOV3/CDDP was insensitive to vitamin K3 compared with the parental cisplatin sensitive SKOV3 cell line. This resistance to vitamin K3 was due to higher levels of p62, a key protein involved in autophagy [69], in SKOV3/CDDP cells compared to SKOV3 cells. Furthermore, the authors determined that vitamin K3 treatment of SKOV3/CDDP cells significantly upregulated NQO1 expression. The activation of NRF2 signalling occurred due to the binding of p62 to KEAP1 that led to an inhibition of NRF2 proteasomal degradation favoring NRF2 translocation into the nuclei activating NQO1 expression. Interestingly, silencing of p62 in SKOV3/CDDP cells treated with vitamin K3 increased apoptosis and downregulated the expression of NRF2 and NQO1. Thus, this study clearly showed that overexpression of p62 in cisplatin-resistant ovarian cancer cell line protects cells from oxidative damage caused by vitamin K3 activating NRF2 signaling, then increasing NQO1 expression [79]. 

Sulforaphane is a dietary isothiocyanate present in various cruciferous vegetables, including broccoli, Brussels sprouts and cauliflower [80,81]. Sulforaphane has several important properties including anti-inflammatory, antioxidant and anti-tumor properties [82,83,84,85]. It has been determined that treatment of A2780 ovarian carcinoma cells with sulforaphane increased apoptosis in a concentration-dependent manner and modulates Glutathione (GSH) and ROS in a time-dependent manner. In fact, sulforaphane treatment significantly decreased ROS production and increased transcription of NRF2 and NQO1 in both A2780 and SKOV3 cell lines. Thus, sulforaphane can modulate NQO1 expression regulating NRF2 signalling pathway [86].

Lingzhi is an edible mushroom (also known as Ganoderma lucidum) widely used as a dietary supplement in traditional Chinese medicine for its presumed health benefits and absence of side effects [87]. Several beneficial effects have been attributed to Lingzhi including anti-cancer, anti-inflammatory and antioxidant effects [88,89,90]. The beneficial effects of Lingzhi are attributed to the presence of many bioactive compounds including triterpenes and vitamins [89]. Hsieh and colleagues investigated the role of Lingzhi extracts treatment in OVCAR-3 ovarian cancer cell line. The authors reported that Lingzhi significantly inhibited cell growth downregulating cyclin D1. Moreover, Lingzhi significantly increased the expression of SOD, catalase, NQO1 and GSTP1 upregulating NRF2 expression, then showing important chemopreventive effects [91].

Resveratrol (3,5,4′-trihydroxy-trans-stilbene) is a natural compound found in grapes with important anti-inflammatory, antioxidant and chemopreventive functions [92,93]. Moreover, resveratrol showed important anti-cancer effects inhibiting tumor initiation, promotion and progression [94,95,96]. An interesting study by Yang and colleagues reported that resveratrol treatment of human ovarian cancer PA-1 cells significantly inhibited cell growth and induced apoptosis. Moreover, resveratrol significantly increased NQO1, Heme oxygenase-1 (HO-1) and p62 gene expression, suggesting a potent antioxidant effect of this compound. Since it has been reported that p62 can bind KEAP1 favoring the translocation of NRF2 into the nucleus [79], it is reasonable to think that this mechanism could also be the reason of the increased expression of NRF2-regulated genes NQO1 and HO-1 found in cells treated with resveratrol [97].

Another interesting study evaluated the therapeutic potential of sulforaphane, curcumin, epigallocatechin gallate, epicatechin, pelargonidin, and resveratrol as anti-cancer agents in OVCAR3, OVCAR5, and SKOV3 ovarian cancer cell lines. The authors determined that, with the exception of epigallocatechin gallate, all other compounds showed an inhibitory effect of ovarian cancer cell lines but induced a significant increase in NQO1 expression, cell cycle arrest and apoptosis. Although epigallocatechin gallate exhibited a higher free radical scavenging activity, it did not induce NQO1 expression. Therefore, the anti-cancer effect is not the same for all antioxidant compounds but depends on the single compound used. Interestingly, the growth inhibitory effect of these compounds does not necessarily require the absence of antioxidant response, since almost all of them increased NQO1 expression even if apoptosis was induced [98].

Along with natural compounds, synthetic compounds also showed important effects in ovarian cancer prevention/progression [84,99]. 

Thiosemicarbazides are an important class of organic compounds with significant pharmacological activities [100,101,102]. In addition, their pharmacological activities can be further improved by glycosylation, a chemical modification that can improve pharmacokinetic and pharmacodynamic properties of many drugs [103]. An interesting study by Czubatka-Bieńkowska and colleagues evaluated the role of thiosemicarbazides and their analogs as anticancer agents in A2780 ovarian cancer cell line. The authors determined that these compounds exert their anti-cancer activity damaging DNA but they do not evoke oxidative stress. Furthermore, they discovered an increased expression of NQO1 gene in response to these compounds, and this effect was specific for the glycosylated S-bond compounds. Thus, the increased NQO1 expression could be a defense response to these compounds, but not sufficient to inhibit apoptosis [104].

Quinones are among the most important drugs used as anticancer agents [105,106]. In fact, these compounds can be found also as natural compounds (e.g., lapachol and β-lapachone) [106,107] and may be used concurrently to chemotherapy or radiotherapy to increase therapy efficiency. The ability of NQO1 to generate cytotoxic hydroquinones can be a good therapeutic strategy to counteract cancer cell proliferation. In fact, an interesting study showed that β-Lapachone specifically induced cell death in cancer cells with elevated endogenous levels of NQO1 [108]. However, a resistance to β-lapachone and other NQO1 bioactivatable drugs has also been reported [3,109]. Thus, chemical modification of these compounds may avoid/delay resistance occurrence. An interesting study evaluated the effects of Selenium-containing quinone-based 1,2,3-triazoles treatment in human ovarian carcinoma cells OVCAR-8 and determined that treatment with these compounds significantly inhibited cancer cell proliferation. Moreover, the authors validated the cytotoxic effect of these compounds in human non-small cell lung adenocarcinoma A549 cell line which constitutively expresses high levels of NQO1 and determined that cell death was NQO1-specific since addition of dicoumarol, an NQO1 inhibitor, spared their lethal effect. The authors explained these results with an inefficient NQO1-dependent redox cycling of these drugs that causes a massive ROS production that leads to apoptosis [110]. In fact, this mechanism has also been reported in other studies [109,111,112]. Therefore, these compounds could be used as novel and efficient anticancer drugs.

The studies discussed in this section are summarized in Table 3.

## 5. Discussion

**Figure 2:**
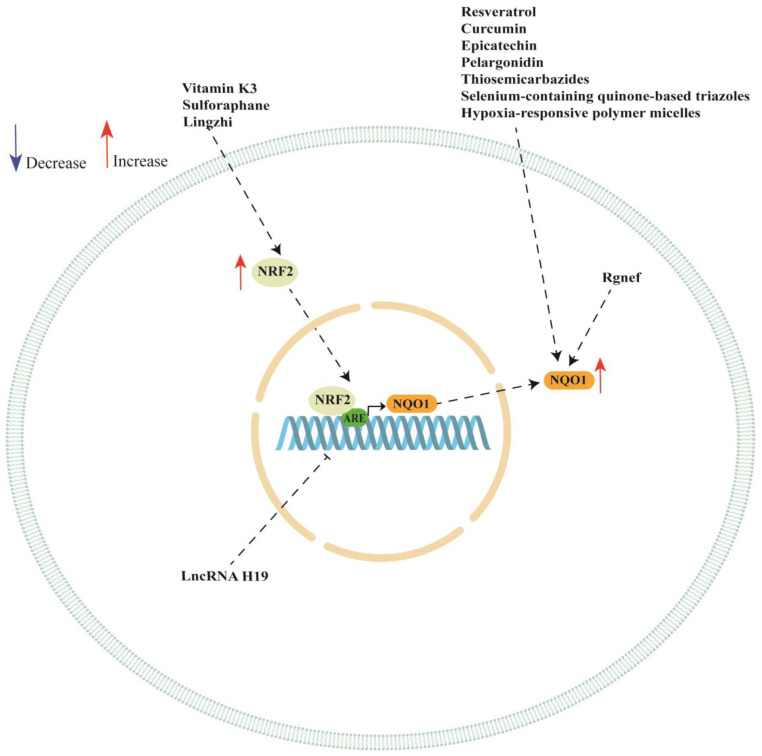
Schematic representation of NQO1 regulation in ovarian cancer cells. ARE: antioxidant response element; NQO1: NAD(P)H quinone dehydrogenase 1; NRF2: nuclear factor erythroid 2-related factor 2.

NQO1 is a promising therapeutic target in ovarian cancer since it plays a pivotal role in ovarian cancer progression and chemotherapy response. In this review, we discussed several studies highlighting the multifaceted role of NQO1 ovarian cancer showing that the expression or activity of this enzyme can be modulated by the presence of SNPs in its gene. Moreover, NQO1 expression can be regarded by long non-coding RNA (such as LncRNA H19), Rho-specific guanine nucleotide exchange factors (such as Rgnef) and hypoxia-responsive polymer micelles (see Table 2). We also showed a key role of NRF/KEAP1 pathway in regulating NQO1 expression in ovarian cancer cell lines. In fact, the analyzed studies reported a key role of vitamin K3, sulforaphane, Lingzhi upregulating NQO1 expression though the activation of NRF/KEAP1 pathway. However, NQO1 expression or activity could also be regulated by other natural and synthetic compounds, although the authors did not investigate the activation of NRF/KEAP1 pathway (see Table 3). A schematic representation of NQO1 modulation is shown in Figure 2. 

Interestingly, although there was an activation of NQO1 and other antioxidant enzymes such as SOD, GSTP1 and HO-1, in ovarian cancer cells treated with these compounds this antioxidant response was not sufficient to avoid cell death. Thus, the increased expression of NQO1 could be a defensive, but inefficient, response of cancer cells to these treatments. These results also explained an important point in antioxidant response. In fact, this clearly shows that an antioxidant response in cancer cells is not always sufficient to avoid cell death since it can be overwhelmed by other cellular processes.

It is important to underline that NQO1 expression is highly activated in cancer cells with mutant Kirsten rat sarcoma viral oncogene homolog (KRAS) signaling [113]. Moreover, mutant KRAS cells show an increased activation of NRF2 (the upstream regulator of NQO1) that upregulates glutamine metabolism [114]. In turn, this allows glutaminase inhibitors to inhibit NRF2/ NQO1 mediated pathways [115,116]. Thus, the use of glutaminase inhibitors such as CB-839 could be a useful therapeutic strategy in cancer cells with high NQO1 expression since they can reduce glutamate availability, creating a metabolic bottleneck.

Although there are not clinical trials evaluating NQO1 modulation in ovarian cancer, two interesting clinical trials showed important results regarding the use of two compounds that can exploit the high expression of NQO1 in tumor cells compared to normal cells. In fact, it has been reported that RH1 (a novel anticancer agent with potent DNA-cross linking activity) is activated within tumors overexpressing NQO1 showing maximal antitumor activity with reduced toxicity in normal tissues [117]. Similarly, ARQ 761 is a β-lapachone analogue that exploits the unique elevation of NQO1 found in solid tumors to cause tumor-specific cell death [118].

Thus, clinical trials are needed to evaluate the role of these two compounds in treating ovarian cancer patients since NQO1 is more expressed in ovarian cancer tissues compared to the normal ones.

In conclusion, we can state that NQO1 modulation plays a key function in regulating cancerous cell response to chemotherapeutic agents. Therefore, the development of therapies targeting NQO1 in ovarian cancer could significantly improve the outcome of this disease.

## Figures and Tables

**Table 1:** NQO1 SNPs in ovarian cancer.

SNP	Alleles	Position
rs1131341	C/A/T	exon 4 of the NQO1 gene
rs1800566	C/T	exon 6 of the NQO1 gene
rs2917666	C/G/T	3′ UTR of the NQO1 gene

**Table 2:** NQO1 cellular modulators in ovarian cancer.

Modulator	Cell Model Used	Results	Reference
Hypoxia-responsive polymer micelles	SKOV3	Hypoxia-responsive polymer micelles synergically act with dicoumarol (a NQO1 inhibitor) reducing NQO1 activity and sensitizing SKOV3 to sorafenib under hypoxia, leading to apoptosis. HIF-1α expression decreased in cells treated with micelles loaded with dicoumarol	[52]
Rgnef	ID8-IP	Knockout of Rgnef in aggressive murine ID8-IP cell line decreased NQO1 expression	[56]
LncRNA H19	A2780 A2780/CDDP	NQO1 expression was higher in A2780/CDDP compared to A2780. Knockdown of lncRNA H19 in A2780/CDDP cells restored cisplatin sensitivity reducing NQO1 and NRF2 expression	[68]
NRF2	A2780 A2780/CDDP	NQO1 and NRF2 expression was higher in cisplatin-resistant A2780/CDDP cells compared to the cisplatin-sensitive A2780 cells. Silencing of NRF2 sensitized A2780/CDDP cells to cisplatin treatment decreasing NQO1 protein expression and increasing cisplatin-induced cell death	[69]

CDDP: Cisplatin.

**Table 3:** NQO1 modulation by natural and synthetic compounds in ovarian cancer.

Modulator	Cell Model Used	Results	Reference
Vitamin K3	SKOV3 SKOV3/CDDP	SKOV3/CDDP were insensitive to Vitamin K3 compared to SKOV3 cell line due to higher levels of p62 in SKOV3/CDDP cells compared to SKOV3 cells. Vitamin K3 treatment of SKOV3/CDDP cells activated NRF2 signalling upregulating NQO1 expression. Silencing of p62 in SKOV3/CDDP cells treated with Vitamin K3 increased apoptosis and downregulated the expression of NRF2 and NQO1.	[79]
Sulforaphane	SKOV3 A2780	Sulforaphane decreased ROS production and increased transcription of NRF2 and NQO1 in both A2780 and SKOV3 cell lines.	[86]
Lingzhi extracts	OVCAR3	Lingzhi extracts inhibited cell growth downregulating cyclin D1 but increased the expression of SOD, catalase, NQO1 and GSTP1 upregulating NRF2 expression	[91]
Resveratrol	PA-1	Resveratrol inhibited cell growth and induced apoptosis but increased NQO-1, HO-1 and p62 gene expression.	[97]
Sulforaphane Curcumin Epicatechin Pelargonidin Resveratrol	OVCAR3 OVCAR5 SKOV3	Sulforaphane, curcumin, epicatechin, pelargonidin and resveratrol inhibited cell growth inducing cell cycle arrest and apoptosis but increased NQO1 expression.	[98]
Thiosemicarbazides	A2780	Thiosemicarbazides and their analogs induce DNA damage without inducing oxidative stress. These compounds increased expression of NQO1 gene.	[104]
Selenium-containing quinone-based 1,2,3-triazoles	OVCAR8	Selenium-containing quinone-based 1,2,3-triazoles treatment of OVCAR-8 cells inhibited cell proliferation probably modulating NQO1 activity.	[110]

CDDP: Cisplatin.

## Data Availability

Not applicable.
